# Positive intergroup contact modulates fusiform gyrus activity to black and white faces

**DOI:** 10.1038/s41598-020-59633-9

**Published:** 2020-02-14

**Authors:** H. Farmer, M. Hewstone, O. Spiegler, H. Morse, A. Saifullah, X. Pan, B. Fell, J. Charlesford, S. Terbeck

**Affiliations:** 1 Institute of Cognitive Neuroscience, UCL, Alexandra House, 17 Queen Square, London, WC1N 3AZ UK; 2 School of Experimental Psychology, University of Oxford, Radcliffe Observatory Quarter, Woodstock Road, Oxford, OX2 6GG UK; 3 School of Psychology, University of Newcastle, University Drive, Callaghan, NSW 2308 Australia; 4 School of Psychology, Plymouth University, Portland Square, Drake Circus, Plymouth, PL4 8AA UK; 5 School of Computer Science, Goldsmiths, University of London, New Cross, London, SE14 6NW UK; 6 School of Psychology, Liverpool John Moores University, Byron Street Campus, Liverpool, L3 3AF UK

**Keywords:** Social neuroscience, Human behaviour

## Abstract

In this study, we investigated the effect of intergroup contact on processing of own- and other-race faces using functional Magnetic Resonance Imaging (fMRI). Previous studies have shown a neural own-race effect with greater BOLD response to own race compared to other race faces. In our study, white participants completed a social-categorization task and an individuation task while viewing the faces of both black and white strangers after having answered questions about their previous experiences with black people. We found that positive contact modulated BOLD activity in the right fusiform gyrus (rFG) and left inferior occipital gyrus (lIOC), regions associated with face processing. Within these regions, higher positive contact was associated with higher activity when processing black, compared to white faces during the social categorisation task. We also found that in both regions a greater amount of individuating experience with black people was associated with greater activation for black vs. white faces in the individuation task. Quantity of contact, implicit racial bias and negatively valenced contact showed no effects. Our findings suggest that positive contact and individuating experience directly modulate processing of out-group faces in the visual cortex, and illustrate that *contact quality* rather than mere familiarity is an important factor in reducing the own race face effect.

## Introduction

Racial prejudice, stereotypes, and discrimination emerge from our tendency to categorise people into in- and out-groups. Racial categorisation can be based on facial features, thus racial biases might begin at face perception. Ito and Urland^1^ suggested that perception of gender, age, and ethnicity occurs at a very early stage of face processing, with differential EEG activation found less than 200 ms after stimulus presentation. A network of limbic and cortical regions – including the amygdala, insula, anterior cingulate cortex, and dorsolateral prefrontal cortex – have previously been associated with intergroup face processing^2–6^. Whilst these regions are predominantly associated with group evaluations and behaviour regulation, the fusiform gyrus has been strongly linked to elements of facial identification including age, race and gender^7–10^. Kanwisher, McDermott and Chun^11^ determined that a region of the fusiform gyrus (FG) selectively responded to human faces. Based on brain lesion studies, Gauthier *et al*.^12^ suggested that the FG was associated with processing faces at an individual level, rather than mere detection of faces per se. In a recent investigation, Koyama *et al*.^13^ examined two brain-damaged patients with bilateral fusiform and parahippocampal lesions. Whilst neither patient showed signs of prosopagnosia, nor an inability to detect faces, their perception of race was selectively impaired, supporting the role of the fusiform gyrus in face identity and social categorisation.

## Processing of Own- and Other-Race Faces

Early behavioural studies found that people are better at recognising faces of own- compared to other-race targets (the own-race bias)^14^. Levin^15^ developed a theoretical model of the own-race effect, suggesting that the own-race advantage is based on perceptual mechanisms, which operate on a categorical level for out-group and on an individual level for in-group faces. He demonstrated that out-group faces are categorised faster by race than in-group faces, suggesting different processing mechanisms for own- and other-race faces. In addition, Sporer^16^ proposed that, due to extensive experience, in-group faces are processed automatically in a configural manner, whilst perception of out-group faces focuses on social categorisation cues. These studies illustrate systematic differences in how own- and other-race faces are processed.

Notwithstanding an apparently automatic effect of target race on face processing, researchers have highlighted that motivational factors can lead people to move beyond category information^17,18^. Hugenberg, Young, Bernstein, and Sacco^19^ proposed that perceptual experience, social categorisation processes, and motivated individuation all contribute (interactively) to the own-race bias. Support for the impact of motivational processes comes from research that created ad hoc minimal groups^20^ in the lab and demonstrated that mere categorisation led to greater recognition of in-group faces despite the absence of previous experience with either novel group^21^. Adducing neuroscience evidence, Van Bavel, Packer and Cunningham^22^ found greater fusiform gyrus activity for in-group compared to out-group faces, when group membership was created using the minimal group paradigm (i.e., different team memberships). Finally, differences in fusiform gyrus activity in response to black and white faces are most pronounced among people with higher implicit race bias, suggesting that top-down motivational processes impact intergroup perception^7^. Because the measure of implicit bias indexes accessibility, this could therefore suggest that intergroup contact could modulate the own-race bias.

## Intergroup Contact and Face Perception

Since Allport’s^23^ proposition that intergroup contact reduces prejudice, numerous studies have demonstrated the proposed relation between contact and prejudice^24^. Contact researchers have further explored the effects of the valence (vs. frequency) of contact, with initial studies finding statistically significant differences between positive and negative contact. For instance, Barlow *et al*.^25^ found that negative contact *increased* prejudice. Relatedly, Paolini, Harwood and Rubin^26^ reported that the category membership of an out-group member was more salient to participants after negative than positive contact. Specifically, references to ethnicity occurred earlier and more frequently when describing the out-group individual after being exposed to negative (vs. positive) non-verbal intergroup behaviour^26^. Whereas prejudice researchers have traditionally considered the effect of contact on explicit prejudice, research has also found evidence of an effect on implicit bias^27^. Considering that the quality of contact may relate to styles of intergroup social cognition, we therefore explore the effect of valence of contact on person-perception specifically. Levin^28^ suggested that individuals with low-frequency intergroup contact might allocate more perceptual resources to social categorisation, and might therefore show lower levels of individuation.

Furthermore, research suggests that individuating experiences do impact face processing. Walker and Tanaka^29^ found that individuals with higher frequency of intergroup contact – thus, arguably, more individuating experience – were more likely to process other-race faces similarly to own-race faces. In other words, time spent interacting with outgroup members mitigated the other-race effect. In addition, Young and Hugenberg^30^ linked motivational and experiential factors, showing that motivation (induced via different task instructions) can reduce the own-race bias when participants had relatively extensive prior experience with individuating out-group faces. Moreover, a lack of experience with out-group members could be overcome when participants were strongly motivated to attend to out-group faces. McGugin, Tanaka, Lebrecht, Tarr, and Gauthier^31^ also found that training to individuate out-group faces led to increased perceptual ability to discriminate such faces. Furthermore, the effect was also transferred to novel faces of the same race. Walker, Silvert, Hewstone and Nobre^32^ employed EEG methods to test the impact of individuating contact, finding that such experiences mediated face-processing effects in early stages of face processing, as reflected in differences in ERPs (i.e., N170 and P400) to other-race faces. These studies illustrate that individuating experiences with outgroups members may indeed have effects on face processing. However, the low spatial resolution of EEG meant that this study was unable to determine exactly which regions of the visual cortex were modulated by intergroup contact.

Previous research has also determined differences in neural activation to black and white faces depending on the specific task (i.e., social categorisation vs. individualisation). For instance, Wheeler and Fiske^33^ determined differences in the amygdala during processing of black and white faces whilst engaging in either social categorisation (i.e., deciding the age of the person) or individuation (i.e., deciding vegetable preferences of the particular person). They found greater amygdala responses to racial out-group members in the categorisation task, but greater amygdala responses to racial in-group faces in the individuation task. In addition, using EEG, Ito and Urland^1^ found larger ERPs (i.e., N170) for own-race faces; however they only found differences when racial categorisation was explicit rather than implicit. Liu *et al*.^34^ argued that responses of the FG to own-race and other-race faces are modulated by task demands (e.g., recognition vs. categorisation). They presented European and Chinese faces to Chinese participants and compared FG activity during a recognition task, which adopted an old / new paradigm and a categorisation task in which participants were asked to indicate whether the face was European or Chinese. They found a significant race by task interaction. For own-race faces, FG responses were equal for the two tasks. Thus, own-race faces were automatically encoded at the individual level regardless of task demands. For other-race faces, however, there was stronger FG activation during the recognition task compared to the categorisation task, suggesting increased processing of out-group faces in the individuation task. Thus, other-race faces were not automatically individuated, but a recognition task motivated individuation.

To our knowledge, the effect of intergroup contact on brain responses using fMRI has not previously been investigated. In the current study we used fMRI to investigate how intergroup contact modulates activity in face-specific visual areas across two different tasks, one which emphasised social categorisation and another which required participants to individuate the faces. Given previous findings that contact valence modulates intergroup biases, and that the own-race effect might be modulated by motivation/attention and prior experience, we predicted that positively-valenced intergroup contact would be associated with greater fusiform gyrus activity for black faces compared to white faces. Additionally, we predicted that individuating experiences would be associated with greater activation during the individuation task. Thus, we predicted that measures of contact quality – but not quantity – would show effects. Finally, since Brosch *et al*.^7^ suggested that the difference in fusiform gyrus activity to black and white faces was most pronounced in individuals with higher implicit bias, we included the race IAT^35^ to additionally assess this effect.

## Methods

### Design

The study used a 2 (ethnicity of faces participants viewed: white, i.e., racial in-group vs. black, i.e., racial out-group) × 2 (task completed while viewing faces: social categorisation vs. individuation) repeated-measures design.

### Participants

Twenty-five participants (mean age = 25.16, *SD* = 4.56, 8 male) took part in this study which was approved by the Plymouth University Faculty Research Ethics Committee. This is a larger study, compared to previous fMRI studies on this topic^33^. All participants gave their informed consent to participate and were paid for their participation. All participants self-identified as being of white ethnicity, were right-handed and were screened for neurological disorders. All research was performed in accordance with guidelines/regulations, as outlined in the University Research Ethics principles.

### Materials

The faces used in the study were taken from the Eberhardt Face Database (EFD) (Mind, Culture, & Society Laboratory at Stanford University, http://www.stanford.edu/group/mcslab/cgi-bin/wordpress/examine-the-research). Sixty white and sixty black faces were selected that were balanced for ratings of attractiveness, racial stereotypicality and age. A further six faces of each ethnicity were selected from the EFD for the training section of the study. Prior to presentation, the chosen stimuli were grey-scaled and resized to 320 × 420 pixels with a white border added to preserve face proportions using a custom MATLAB script^36^. They were then equalised for luminance and contrast using the SHINE toolbox^37^. Finally, scrambled versions of each face were created for use in the non-face control condition.

### Procedure

#### Pre-task questionnaires

Prior to taking part in the studies participants were selected from a larger pool of respondents who had completed an online survey which contained a series of questions relating to their contact with black people. The questionnaires included measures of:

- Contact quantity. Contact quantity was measured using five items, including items such as “How much contact do you have with black people as neighbours?” (1 = *None at all* to 5 = *A great deal*.). This was based on the items used in previous studies^38^ (α = 0.86 in Van Dick *et al*.^38^ and α = 0.73 in this study).
- Contact quality. Contact quality was measured using four items (a fifth item was removed to improve the measure’s reliability), including items such as “To what extent did you experience the contact with black people as superficial or intimate?” (1 = *very superficial* to 5 = *very intimate*) based on items previously used^39^ (α = 0.75 in previous studies, α = 0.81 in this study).
- Cross group friendship. One item asked “How many close friends do you have, who are black?”^40^.
- Valenced contact. A two-item scale measured positive and negative contact, respectively. This differs from the contact quality measure in that it assesses the frequency/quantity of positive and negative contact separately: *“On average, how frequently do you have NEGATIVE/BAD contact with Black people”* and *“On average, how frequently do you have POSITIVE/GOOD contact with Black people?”* (1 = *never* to 7 = *extremely frequently*)^26^. (*r* = −0.25, *p = *0.21).
- Individuating experience. A ten-item scale included items such as *“How often do you have Black friends round to your place?”* (1 = *Never* to 5 = *Frequently*), developed by Walker *et al*.^32^. (α = 0.94 in the original study and α = 0.92 in this study). The measure of individuating experience was developed for “…specifically asking about contact that was more linked to individuation via prosocial behavior, empathy, cooperation and self-disclosure.” (Walker *et al*.^32^ p. 18).

These scores, obtained during the recruitment phase, were then used to select a sample of participants with a varied range of contact experiences. This was determined on the basis of the individuating experience measure^32^; this measure was chosen because the range of responses was the closest to the average range of responses of all measures (based on z-scored data). Participants with an individuated contact score below 20 were categorised as low, those with an individuated contact score between 20–30 were categorised as medium, and those with a score between 30–50 were categorised as high. Note that this procedure was only used as an approximation to select a diverse sample of participants in terms of their contact experience (i.e., in order to avoid having participants who all reported very high or very low contact); the categories were not used during data analysis. Instead we used regression to examine the effect of individual differences in contact, measured as continuous scores, on BOLD response. Twenty-five participants (9 high, 6 medium, 10 low), were invited to the scan (see Table 1 for group means and standard deviations). All measures were significantly correlated (See Table 2), however multicollinearity calculations showed low factors (i.e., all Variance Inflation Factor (VIF) scores around 1–2, with the highest being 3.8) suggesting at most only moderate correlation between the different constructs measured.

**Table 1:** Means and standard deviations (SD) for the various measures of intergroup contact and for the IAT result.

Measure	Low: N = 10 Mean (SD)	Medium: N = 5 Mean (SD)	High: N = 10 Mean (SD)
Contact Quantity (Range = 8–31)	11.80 (2.78)	18.60 (2.30)	21.70 (4.85)
Contact Quality (Range = 10–28)	21.00 (4.50)	22.40 (3.00)	25.20 (2.57)
Cross-Group Friendship (Range = 1–4)	1.20 (0.42)	2.60 (0.55)	3.30 (0.48)
Negative Contact (Range = 1–5)	2.00 (1.15)	2.40 (1.14)	2.20 (1.14)
Positive Contact (Range = 3–7)	4.50 (1.27)	5.40 (0.90)	6.10 (1.12)
Individuating Experiences (Range = 12–47)	19.00 (4.87)	29.80 (4.87)	37.00 (6.84)
IAT	0.33 (0.33)	0.13 (0.19)	0.26 (0.38)

**Table 2:** Correlations between the contact scores (measures were all z-transformed to normalise).

	Contact quality	Cross-group friendship	Negative contact	Positive contact	Individuating experience
Contact quantity	0.55**	0.79**	−0.16	0.56**	0.77**
Contact quality		0.41*	0.00	0.54**	0.60**
Cross-group friendship			0.25	0.53**	0.76**
Negative contact				−0.26	−0.07
Positive contact					0.69**

**Correlation is significant at the 0.01 level (2-tailed).

*Correlation is significant at the 0.05 level (2-tailed).

#### Experimental task before and during the fMRI scan

Participants completed two main tasks in this study that were split into separate blocks within the scanner; these tasks were based on tasks previously used by Wheeler and Fiske^33^. The first of these was an individuation task which required participants to carry out a learning phase outside the scanner in which they learned the favourite vegetable of each of the people represented by the faces. This learning task involved first seeing each face with a label for their favourite vegetable displayed below. After all faces had been presented the entire set of faces and labels was repeated. Participants were then presented with two options for each face’s favourite vegetable displayed on the bottom right and left of the screen and had to press a key to indicate which was correct. They then received feedback on their performance. During the scanning session, they again had to choose between two options but received no feedback on their choice.

The second main task participants completed was a social categorisation task in which they had to decide whether each of the faces was over or under 25 years old. For this task participants did not receive previous training on which of the two options was correct for each face and no feedback was given during the task itself. An additional no-face control task was also used to identify face-specific areas of activation. In this task participants viewed the scrambled-face images, each of which had either a triangle or a circle superimposed on to it. Participants had to indicate whether the shape on the scrambled image was either a triangle or a circle. Prior to entering the scanner participants completed a practice version of the task in which they first learned the vegetable preferences for 12 faces (6 black, 6 white). These faces were not used in the main task. They then completed a short block of both the individuation and social categorisation tasks in which they saw all faces (blocks were counterbalanced across participants); in each of these short blocks there were also three trials of the no-face control task. Finally, they learned the vegetable preferences of all 120 faces to be seen in the scanner. Before entering the scanner participants also completed a version of the race (black vs. white) IAT which was administered using the FreeIAT software^41^ and scored using the improved algorithm recommended by Greenwald *et al*.^35^.

**Figure 1:**
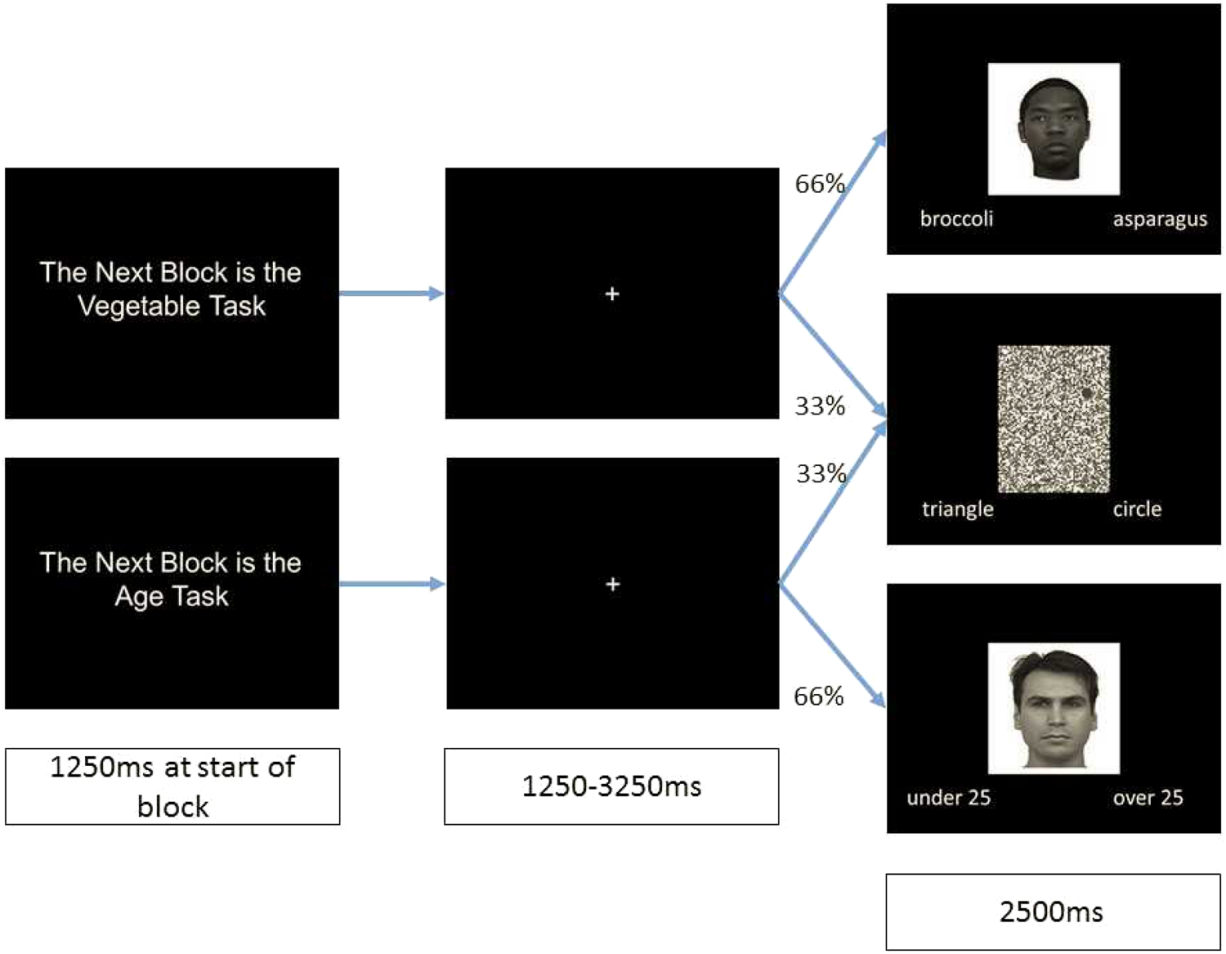
Direct link: http://wiki.cnbc.cmu.edu/Face_Place. Method for scanner session. At the start of each block participants were informed of the task for that block. They then saw 90 trials, two thirds of which were task-relevant faces and one third of which were no-face control trials. Example pictures of black and white faces taken from the Tarrlab’s FacePlace database: Stimulus images courtesy of Michael J. Tarr, Center for the Neural Basis of Cognition and Department of Psychology, Carnegie Mellon University, http://www.tarrlab.org/. Funding provided by NSF award 0339122.

Once inside the scanner participants carried out four blocks of trials alternating between the individuation task (favourite vegetables) and the social categorisation task (above or under 25 years). The order of tasks was counterbalanced across participants. Each block consisted of 60 trials of the main task of which 30 involved white faces and the other 30 black faces; each block also contained an additional 30 trials of the no-face control task. These 90 trials were presented in a random order. Each block started with a screen informing participants which task the block required. Then each trial was preceded by an inter-stimulus interval (ISI) in which a fixation cross appeared for between 1.25 and 3.25 seconds. The variation in ISI timing achieved an effective temporal sampling resolution much finer than one TR for each of these periods. The lengths of the intervals were uniformly distributed for each period, ensuring that Evoked Haemodynamic responses (EHRs) time-locked to the events were sampled evenly across the trials. In each trial participants had 2.5 seconds to respond (see Fig. 1).

#### Image acquisition and data analysis

A 1.5 T Siemens TIM Avanto scanner with 32-channel head coil was used to acquire both T1-weighted structural images and T2*-weighted echoplanar images (64 × 64 pixels; 2.5 × 2.5 mm; echo time, 50 ms) with blood oxygen level-dependent (BOLD) contrast. Each volume comprised 35 axial slices (2.5 mm thick, oriented approximately to the anterior commissure posterior commissure plane), covering most of the brain but omitting inferior portions of the cerebellum. Functional scans were acquired in one session comprising 610 volumes (~30.15 min). Volumes were acquired continuously with an effective repetition time of 3 seconds per volume. The first four volumes of the scan were discarded to allow for T1 equilibration effects. Following the functional scanning, a 6 min T1-weighted MPRAGE structural scan was collected at a resolution of 1 × 1 × 1 mm. Stimuli were projected onto a screen behind the participant and viewed in a mirror positioned over the participant’s head and participants responded using a 4-button response box. All stimuli were presented with Cogent running under Matlab 2014b permitting synchronisation with the scanner and accurate timing of stimuli presentation. Behavioural data analysis was performed offline, and event timings were prepared for subsequent general linear model (GLM) analyses of fMRI data.

Data were processed and analysed using SPM12 (www.fil.ion.ucl.ac.uk/spm). The EPI images of each participant were realigned to a mean EPI image for that participant. Images in which the participant moved more than 1.5 mm or had rotation of more than 1 degree were visually examined and if seen to contain artefacts were removed from the analysis and replaced with volumes interpolated from the preceding and subsequent images. No participant had artefacts in more than 5% of images. Each participant’s structural image was processed using a unified segmentation procedure combining segmentation, bias correction, and spatial normalization to the MNI template^42^. The same normalization parameters were then used to normalize the EPI images. Finally, the images were spatially smoothed in order to conform to the assumptions of the GLM implemented in SPM12 by applying a Gaussian kernel of 8 mm FWHM.

A first-level GLM was created for factorial analyses. This GLM modelled BOLD activation and only used data from the face trials and had a 2 × 2 model of racial group (white/black) × task (individuation/social categorisation). The four conditions (white individuation, white social categorisation, black individuation and black social categorisation) were modelled as separate regressors with onsets at the start of the choice period. Four regressors of no interest modelled activity during the no-face control task, one for each combination of the task performed in that block and the race of scrambled face. In addition, trials in which the participant did not choose a picture were modelled with a separate regressor. The residual effects of head motion were modelled as covariates of no interest in the analysis by including the six head-motion parameters estimated during realignment. The time points of volumes that had been found to contain artefacts were modelled as additional covariates of no interest. Contrasts derived from the analysis of variance (ANOVA) model were thresholded at *p < *0.05 FWE corrected. Anatomical Regions were determined using the AICHA atlas^43^.

In addition to the frequentist analyses reported below we also conducted additional Bayesian analyses using JASP (JASP Team, 2019^44^) which we report in our supplementary materials.

## Results

### Behavioural results

#### Reaction time and accuracy analysis

A JZS Bayes factor ANOVA^45^ with default prior scales revealed that the main effects model was preferred to the interaction model by a Bayes factor of 2.84. For a complete Bayes analysis of the behavioural and fMRI data please see Supplementary file.

In order to examine whether the social categorisation versus individuation task led to distinct behavioural results for black vs. white faces, two 2 × 2 JZS Bayes factor ANOVAs with default prior scales were carried out with race (black vs. white) and task (individuation vs. social categorisation) as the independent variables. The first ANOVA used reaction time as the dependent variable, while the second used accuracy. The ANOVA on reaction times revealed a non-significant effect of task, *F*(1, 24) = 3.66, *p* = 0.068, η^2^_p_ = 0.16. There was a significant main effect of race, *F*(1, 24) = 4.43, *p* = 0.046, η^2^_p_ = 0.16. Participants were faster to respond to white (Estimated Marginal Mean (EMM) = 1690 ms, Mean Standard Error (MSE) = 30 ms) than to black (EMM = 1710 ms, MSE = 40 ms) faces. The interaction between task and race was also significant, *F*(1, 24) = 7.82, *p = *0.01, η^2^_p_ = 0.25. Pairwise comparisons indicated that this interaction was driven by significantly faster RTs for white (*M* = 1630 ms, SE = 30 ms) compared to black (*M* = 1690 ms, *SE* = 20 ms) faces in the social categorisation task, *t*(24) = 3.76, *p* = 0.001, but not in the individuation task (white: *M* = 1730 ms, *SE* = 40 ms; black: *M* = 1730 ms, *SE* = 50 ms), *t*(24) = 0.14, *p* = 0.893.

The ANOVA on accuracy revealed a significant effect of task, *F*(1, 24) = 104.25, *p < *0.001, η^2^_p_ = 0.81. Overall participants were more accurate during the social categorisation task (EMM = 68.47%, MSE = 1.65%) than during the individuation task (EMM = 51.17%, MSE = 1.23%). There was also a significant main effect of race, *F*(1, 24) = 4.69, *p* = 0.041, η^2^_p_ = 0.16. Participants were more accurate for white (EMM = 60.83%, MSE = 1.19%) than for black (EMM = 58.8%, MSE = 1.35%) faces. The interaction between task and race was non-significant, *F*(1, 24) = 3.90, *p* = 0.06, η^2^_p_ = 0.14.

#### Relationship between behavioural results and contact measures

Next, we conducted a series of regressions to investigate whether behavioural responses to white and black faces in our two tasks were predicted by our contact variables. We first created a measure of race effect for both reaction time and accuracy scores by subtracting performance on black faces from performance for white faces separately for each task condition. We then ran four linear regressions with the race effect in reaction time and accuracy in each task as the dependent variable and IAT scores, measures of individuating experience, quality, quantity, friendship, and the positive and negative contact scales included as predictors. None of the models produced significant results (See Table 3).

**Table 3:** Measure, task condition, adjusted R^2^, *F* statistic and significance value for each of the four regressions between contact variables and behavioural data.

Measure	Task	Adjusted R^2^	F	p
Reaction Time	Individuation	0.174	1.72	0.17
Reaction Time	Categorisation	0.121	1.47	0.242
Accuracy	Individuation	0.023	1.08	0.416
Accuracy	Categorisation	−0.238	0.34	0.924

#### Relationship between contact measures and implicit bias

In order to examine the relationship between our measures of contact and participants’ implicit racial bias we conducted a further linear regression with our contact measures entered as predictors and IAT score entered as the dependent variable. This model found no significant relationship between contact scores and implicit bias, R^2^ = −0.037, *F*(6,18) = 0.86, *p* = 0.544.

### fMRI Results

#### Whole brain main effects and interactions

**Figure 2:**
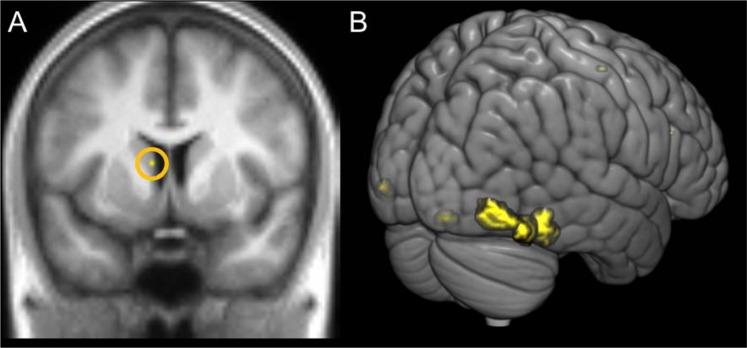
Whole brain FWE corrected activations. (**A**) Caudate nucleus activation revealed in the White Face > Black Face contast. (**B**) Areas activated in the Face > Scrambled contrast.

In order to investigate the effects of race and task on whole brain activation a series of contrasts were run on the second level GLM examining the effect of race of face (white vs. black), task (individuation vs. social categorisation) and the interaction between them. The only significant FWE corrected result was in the white > black contrast which revealed that viewing white faces as opposed to black faces led to significantly greater activation of the left caudate nucleus (see Table 4 and Fig. 2A). To identify brain areas that were selectively activated by faces, a contrast of all face conditions vs. all scrambled conditions was carried out. This revealed a significant voxel level FWE corrected activation centred on the right fusiform gyrus (rFG) as well as on the left inferior occipital gyrus (lIOG), the left lateral occipital gyrus (lLOG), the left supplementary motor cortex (lSMA), and the right inferior frontal sulcus (rIFS) (see Table 4 and Fig. 2B).

**Table 4:** Peak voxel coordinates in MNI space, z-values and cluster sizes for analyses showing significant effects at voxel level FWE corrected significance of 0.05. Contrasts that did not show any significant activations are not listed.

Region (BA)	Hem.	X	Y	Z	Z-Score	Cluster Size
White>Black						
Caudate Nucleus	L	−8	6	10	5.00	2
Face>Scrambled						
Fusiform Gyrus (37)	R	38	−42	−20	6.39	548
Inferior Occipital Gyrus (37)	R	42	−58	−18	6.20	
Inferior Occipital Gyrus (37)	L	−38	−50	−26	5.44	41
Lateral Occipital Gyrus (19)	L	−42	−86	−6	5.39	28
Supplementary Motor Area (32)	L	−6	18	48	4.93	3
Inferior Frontal Sulcus (48)	R	42	28	22	4.88	1

#### Face specific region of interest analysis

In order to examine how variations in the amount and nature of contact with black people was associated with our white participants’ responses to black faces within the visual cortex an additional region of interest (ROI) analysis was conducted on the three clusters in the occipital cortex identified in the face > scrambled contrast using MarsBar^46^. Cluster wide parameter estimates from the contrasts between race (black > white) and task (categorisation > individuation) and the interaction between race and task ((black categorisation > white individuation) > (black categorisation > white individuation)) were extracted from each of the ROIs. The parameter estimates for each set of contrasts were then entered into three multivariate-multiple regression models with participants’ scores from the IAT, the measure of individuating experience, quality, quantity, friendship, and the positive and negative contact scales included as predictors (see Table 5 for R^2^, *F* statistic and *p* value of all regression models in each region).

**Table 5:** Cluster, contrast, adjusted R^2^, *F* statistic and significance value for each of the three multivariate regressions between contact variables and ROI data.

Contrast	Cluster	Adjusted R^2^	F	P
Race	rFG	−0.204	0.42	0.877
	lIOG	−0.171	0.5	0.822
	lLOG	−0.306	0.197	0.982
Task	rFG	0.096	1.36	0.282
	lIOG	−0.011	0.96	0.488
	lLOG	0.001	1.01	0.462
Interaction	rFG	0.326	2.66	0.047*
	lIOG	0.354	2.88	0.035*
	lLOG	0.093	1.35	0.288

* indicates significant at *p < *0.05.

**Figure 3:**
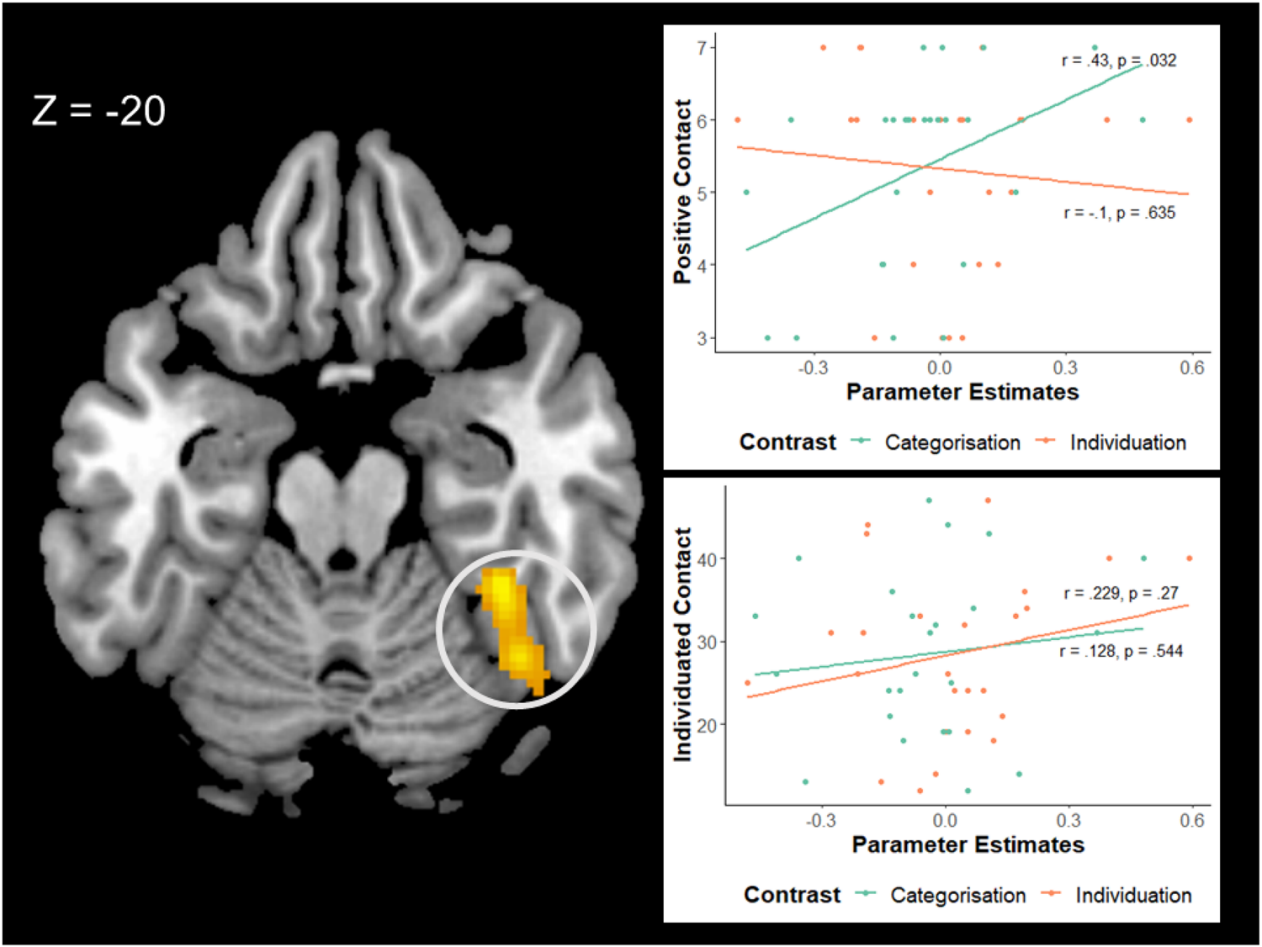
Correlation between parameter estimates extracted from rFG and the standardized positive contact and individuated contact scales. We have represented the Task x Race interaction by plotting the parameter estimates for each of the task contrasts separately with the Black Categorisation - White Categorisation in green and the Black Individuation - White Individuation in orange. Higher values on the y axis represent greater scores on each scale while values further to the right on the x axis represent greater activation for black faces relative to white faces. Annotations show Pearson’s r and associated p value for each correlation.

Of the three multivariate regressions only the one examining the interaction contrasts produced significant results. We here first report the results for each of the three clusters seperately. For the rFG, the regression produced a signficant model, adjusted R^2^ = 0.33, *F*(7,17) = 2.57, *p* = 0.047 (see Table 5 for R^2^, *F* statistic and *p* values of all regressions). Within this model, positive contact positively predicted the size of parameter estimates extracted from the rFG, while individuating experience negatively predicted the size of parameter estimates (see Table 6 and Fig. 3). Examination of the slopes from the black categorisation-white categorisation and black individuation-white individuation contrasts showed that higher positive contact scores correlated with greater activation for black faces compared to white in the categorisation task, but lower activation for black faces compared to white faces in the individuation task. By contrast, higher individuating experience scores correlated with greater activation for black faces compared to white faces in the individuation task and the reverse in the categorisation task.

**Table 6:** Summary of regression analysis for measures predicting size of parameter estimates extracted from the rFG interaction contrast along with Pearson’s r.

Measure	β	p	R
Positive contact	0.181	0.009**	0.363
Negative contact	−0.077	0.162	−0.346
Implicit attitude (IAT)	−0.168	0.378	0.018
Individuating contact	−0.024	0.037*	−0.075
Quantity of contact	−0.021	0.264	−0.051
Quality of contact	−0.007	0.731	−0.083
Cross-group friendship	0.175	0.080	0.105

* indicates significant at *p < *0.05, ** indicates significant at *p < *0.01.

**Figure 4:**
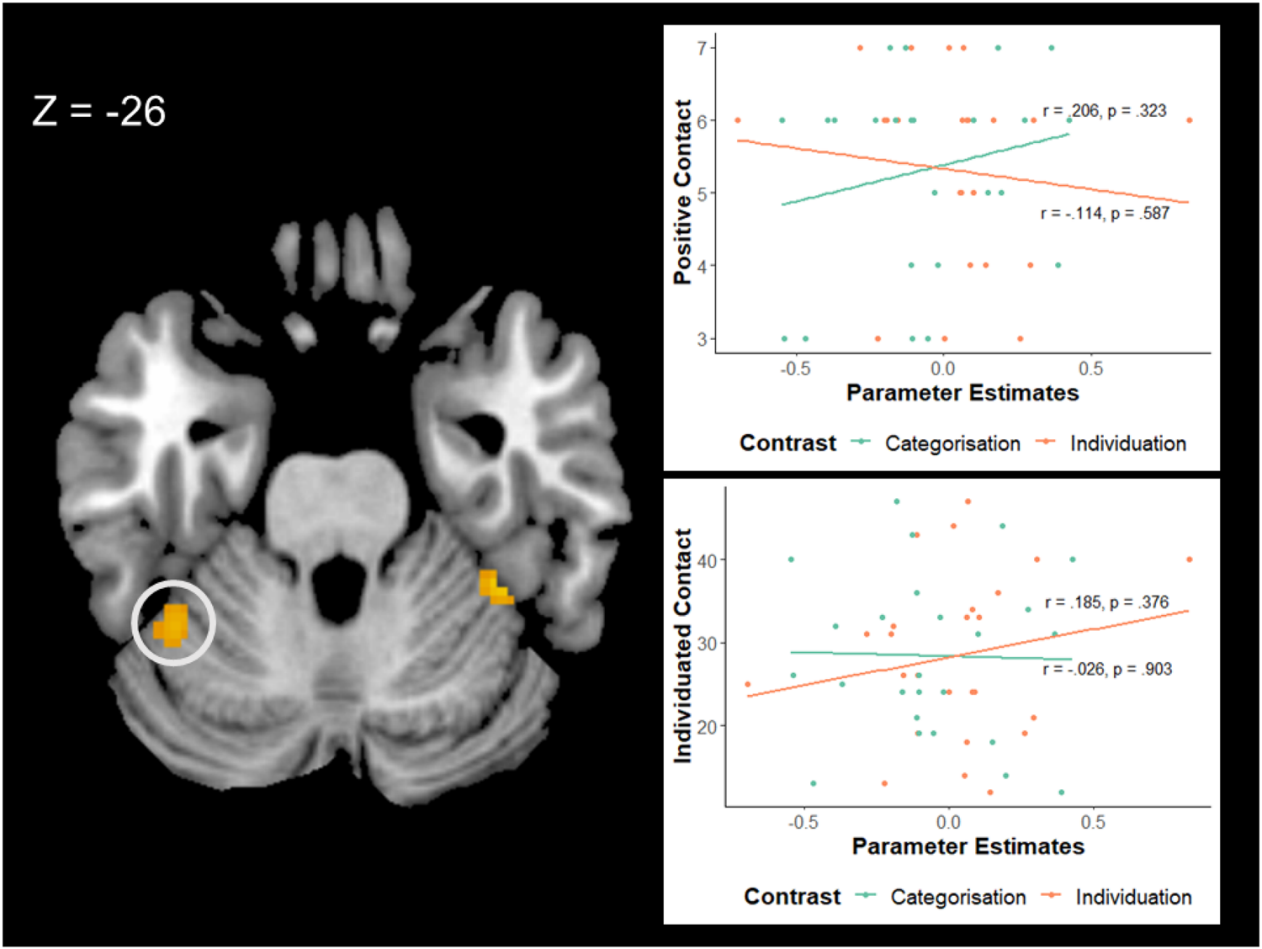
Correlation between parameter estimates extracted from the left inferior occipital gyrus and the standardized positive contact and individuated contact scales. We have represented the Task x Race interaction by plotting the parameter estimates for each of the task contrasts separately with the Black Categorisation - White Categorisation in green and the Black Individuation - White Individuation in orange. Higher values on the y axis represent greater scores on each scale while values further to the right on the x axis represent greater activation for black faces relative to white faces. Annotations show Pearson’s r and associated p value for each correlation.

A significant model was also found for the lIOG ROI, adjusted R^2^ = 0.354, *F*(7,17) = 2.88, *p* = 0.035. Within this model positive contact positively predicted the size of parameter estimates, while individuating experience negatively predicted the size of parameter estimates extracted from the lIOG (see Table 7 and Fig. 4). Examination of the slopes from the black categorisation-white categorisation and the black individuation-white individuation contrasts showed that higher positive contact scores correlated with greater activation for black faces compared to white faces in the categorisation task, but lower activation for black faces compared to white faces in the individuation task. In contrast, higher individuating experience scores correlated with greater activation for black faces compared to white faces in the individuation task, but lower activation for black faces compared to white faces in the categorisation task.

**Table 7:** Summary of regression analysis for measures predicting size of parameter estimates extracted from the lIOC. along with Pearson’s r.

Measure	β	p	R
Positive contact	0.186	0.007**	0.281
Negative contact	−0.054	0.316	−0.289
Implicit attitude (IAT)	−0.151	0.425	0.082
Individuating contact	−0.031	0.001**	−0.183
Quantity of contact	−0.008	0.65	−0.061
Quality of contact	−0.017	0.374	−0.207
Cross-group friendship	0.172	0.080	0.076

** indicates significant at *p < *0.01.

For the lLOC postive contact postively predicted the size of parameter estimates, *β* = 0.151, *p = *0.021, r = -363. However the overall model was non-signficant, adjusted R^2^ = 0.093, *F*(7,17) = 1.35, *p* = 0.287 and no other predictors were signficant.

To determine the extent to which our predictors contributed to the overall model we followed Fox and Weisberg’s^44^ method and ran a Type II MANOVA to examine the contribution of each predictor to the full multivariate model. This analysis revealed that of the 7 predictors only positive contact signficantly predicted activation across all three ROI’s, Pillai’s Trace = 0.44, F(7,17) = 4.01, *p* = 0.028. By contrast the predicitve power of individuating contact on the total model was marginally signficiant, Pillai’s Trace = 0.35, F(7,17) = 2.69, *p* = 0.083.

## Discussion

In this study, we investigated how intergroup contact is associated with activation of neural regions involved in the processing of black and white faces across two different tasks, one which emphasised social categorisation and another which required participants to individuate the faces. Our study revealed three effects of interest concerning a region activated more by white than black faces, occipital regions activated more by black or white faces compared to scrambled faces, and the associations between different types of intergroup contact and neural activity in these face-relevant occipital areas as a function of task type. Specifically, more positive contact was associated with higher rFG activity when processing black compared to white faces during the social categorisation task. In addition, greater individuating experience was associated with greater rFG activation for black vs. white faces in the individuation task.

First, using whole brain analysis, we examined the effects of race of face, as well as task (i.e., individuation versus categorisation). Regardless of task, we found that white faces elicited greater activation in the caudate nucleus than black faces, a region which has previously been strongly related to reward processing. For example, Lauwereyns *et al*.^47^ used single neuron recordings to show that caudate neurons participate in the anticipation of visual stimuli that predicted reward in monkeys. While in humans, Delgado, Stenger and Fiez^48^ determined that reward related activation in the caudate nucleus was associated with different levels of motivation in a guessing task. The caudate has also been implicated in the processing of faces, particularly emotional faces^43^. It is possible that the caudate nucleus findings in the current study relate to participants finding the task more rewarding/motivating for white faces as opposed to for black faces.

Second, we found that processing faces of either race, as opposed to scrambled faces, led to activity in occipital regions including the rFG, the lIOG and the lLOG. These results were consistent with previous research, revealing a well-established network for face processing^7–10^.

Third, and most interestingly, we performed three mutlivariate regression analyses, one each for task, race and their interaction, with our occipital activity as the dependent variables and our measures of intergroup contact and implicit bias as our predictors. While we did not find any relationship between our predictors and the contast derived from race and task, the analysis indictated that across all three regions BOLD response to black faces compared to white faces, when performing the social categorisation task vs. the individuation task, was positively correlated with positive contact. and negatively correlated with individuating experiences. In other words, greater positive intergroup contact was associated with higher for black faces compared to white faces during social categorisation in regions involved in the processing of faces. We also found evidence that greater individuating experience with black individuals was associated with higher activation for black faces compared to white faces during individuation in two of our three ROIs, the right fusiform gyrus and the left inferior occipital gyrus, an area also associated with face processing and facial identity^49^. However, this effect was not significant when controlling for covariance across models.

Since in previous fMRI studies^34^ the other-race-effect has been linked to reduced BOLD signal when processing out-group faces, this pattern suggests that, depending on task demands, both more frequent positive contact and greater individuating experiences can be associated with greater neural processing of outgroup faces and so reduce the strength of the other-race-effect. The measures of negative contact, contact quantity, contact quality and cross-group friendship did not yield any reliable effects and nor did level of implicit racial bias as measured by the IAT.

Our results suggest that the valence of experienced intergroup contact is associated with facial processing, and that positive contact is associated with increased neural activity when processing out-group faces during social categorisation. These findings support the theory that factors other than simply the quantity of intergroup contact (specifically, the valence or intimacy of that contact) might underlie the own-race effect (i.e., greater processing of own-race faces). Given that contact quantity equated to mere experience with out-group faces, whilst contact valence relates to the quality of the contact, our study is consistent with the view that the own-race effect is not determined simply by frequency of experiences or familiarity^50^.

Our study further illustrates the fact that observed differences in face perception for own-race and other-race are not inevitable, and that the specific task being used as well as the observer’s previous history of intergroup contact can both modulate neural activation underlying face perception. This finding is in line with previous research which also found differences in the neural processing of other race faces depending on social categorisation and individuation^1,33^. Thus the current study provides further evidence that higher level task demands can have an effect on racial face processing in the brain even within the visual cortex.

Our results showed an interaction between race, task and contact measures such that more frequent positive contact predicted increased response to black vs white faces in the social categorisation task. To a less extent we also found evidence that individuating experiences predicted differences in BOLD response in face specific visual areas when processing white and black faces during the individuation task. This might be explained by the fact that multiple positive encounters with different individuals of another race could change social categorisation for the out-group as a whole, whilst contact with isolated individuals from the out-group modulates facial processing only when individuation between members of the out-group is task relevant. Indeed, prior research argues that change is more significant when perceivers encounter multiple out-group members who are still categorised as typical members of that out-group, rather than merely one person, who does not represent the group stereotype and can be sub-typed, as non-representative^46^. Thus, to summarise, regarding the regression effects, we found that:

- Having frequent positive contact with black people increased activation for black faces in the social categorisation task; this suggests that previously observed increased activation for in-group faces during categorisation can be altered, leading to increased activation when processing out-group faces.
- To a lesser extent we found evidence that having individuating experience with black people (that is, contact that is specifically linked to, e.g., seeing the person as an individual) enhanced the neural processing for black faces when having to classify them individually (in the individuation task). This suggests that individuals can learn to perceive a person as an individual rather than merely as a member of a social group.

The neural effects in the visual cortex did not translate to behavioural effects, as we also expected them to be relatively small, given that the behavioural tasks were not particularly demanding. In addition, the whole brain analysis did not identify areas that showed an interaction in activity between the two tasks which has been found in previous research^1,34^. One possible explanation for the lack of this effect is that, unlike previous studies, our study explicitly sought to recruit participants who had a range of levels of contact with black people. Since we show in our linear regression analysis that factors relating to contact play a modulating role in the activity of regions involved in processing faces of in- and out-group races, it is possible that we did not find areas showing a race by task interaction in our group analysis due to the wider variation in familiarity and interaction with black participants among our sample when compared to previous studies. Furthermore, we did not find an effect of negative contact, which might be due to the fact that very few participants reported frequent negative contact, which is consistent with, for example Barlow *et al*.^25^ and Paolini, Harwood and Rubin^26^; thus, compared to positive contact, the limited variance between people in reported negative contact might have been responsible for this effect (*M*_negative_contact = _2.16, *SD* = 1.10; *M*_postive contact_ = 5.32, *SD* = 1.3).

One limitation of the current study is the fact that we have a relatively small sample size for detecting correlations with individual differences^47^. However, we note that the reliability of the results is increased due to our study being designed in a manner that increases power within subject by using a large number of repetitions (60 trials with a total time of 4.75 mins per condition) for each cell of our design which recent work suggests is sufficient to significantly boost replicability even with relatively small sample sizes^51^. In addition to our high level of within-subject sampling we focused our ROI analysis on areas that were both theoretically justified and identified empirically with contrasts that were orthogonal to those used to examine the relationship between neural activation and individual differences. To examine this issue we used GPower^52^ to run a post-hoc test of power for our linear regressions; this showed that our power to detect a significant effect in a regression model with 7 predictors, alpha set at 0.05 and an effect size calculated with an R2 of 0.5 (our significant regressions had R2 values of 0.54 and 0.52) was 0.9, which is well above the conventional cut off of 0.8. However, despite these caveats future work will be needed to replicate these effects and test them in larger samples. We also note that the contact measures were highly related and theoretically similar, but did not all show the same pattern. For instance, it is still not clear at a theoretical level why positive contact, but not friendship, would show this pattern. One could speculate that a single individual friendship might not generalise as strongly to the group as a whole as a measure of overall positive contact. Finally, the contact measures failed to predict any variance in either reaction time or accuracy means and thus the effects should be interpreted with caution.

As is the case with all contact studies using self-reports of contact, a second limitation to our study is that we retrospectively assessed the level of contact based, although self-reports of contact have been validated with observer reports^53^. At worst, results may indicate the effect of *perceived* frequency and valence of contact on neurological intergroup phenomena – a useful question in its own right. This question of whether personal beliefs and recollection of interactions with outgroup members is germane to face processing remains relevant, especially given that such cognitive phenomena – though potentially reconstructed – are likely to themselves be caused by more strictly objective phenomena such as local demographics and actual contact experience^54–56^. Future research could more directly test the hypothesis that (objective) intergroup contact modulates other-race face processing, by experimentally inducing intergroup contact of either positive or negative valence and investigating its immediate effects on the neural basis of race face processing. Such research will require a balance of ecological validity and experimental control that exceeded the scope of this early-stage enquiry.

A final limitation of our study is that, due to demands of scanning time, we identified regions responding to face using a contrast between the faces in our study and scrambled faces. It is possible that the use of an independent face-localiser would have allowed us to detect more focal face specific regions and increased our power to detect areas that showed differences in their processing of same and other race faces and in their sensitivity to the effects of contact. Future research in this area could use more focal localizers to identify more specific areas that respond differently to both race and task.

In conclusion our study indicates that both the valence of intergroup contact and the amount of individuating experience with a racial outgroup were associated with differential racial face processing, and thus contact valence may help to overcome racial biases in the processing of out-group faces that occur at the neural level.

## Supplementary information

3files.
