# Supplementary material for: Positive intergroup contact modulates fusiform gyrus activity to black and white faces
Source: Sci Rep. 2020 Feb 14;10:2700. doi: 10.1038/s41598-020-59633-9 (PMC7021708; doi:10.1038/s41598-020-59633-9)
Supplement: Supplementary file 1 — 3files. [file 41598_2020_59633_MOESM1_ESM.docx]

Positive intergroup contact modulates fusiform gyrus activity to black and white faces: Supplementary Materials

# Supplementary Bayesian Results

Below we report an alternative series of analyses carried out using a Bayesian framework as opposed to the more traditional Frequentist framework. The use of this Bayesian framework has an advantage when considering the problem of the large number of multiple comparisons made in the models used in our study as many of the issues surrounding the increased risk of a type one error due to increased resolve within a Bayesian framework ^1,2^.

### Behavioural Results

#### Reaction time and accuracy analysis

In order to examine whether the social categorisation versus individuation task led to distinct behavioural results for black vs. white faces, two 2 x 2 repeated measures Bayesian ANOVAs with default prior scales ^3^ were carried out with race (black vs. white) and task (individuation vs. social categorisation) as the independent variables. The first ANOVA used reaction time as the dependent variable, while the second used accuracy. The ANOVA on reaction time revealed moderate evidence that the task only model was preferred to the null, Bayes Factor in favour of the alternative hypothesis (BF_10_) = 9.58 and moderate evidence that the task only model was preferred to the full factorial model, Bayes Factor (BF) = 4.96. Comparing effects across matched models revealed moderate evidence for the inclusion of the task effect, Bayes Factor in favour of inclusion (BF_inclusion_) = 9.52, but anecdotal evidence against the inclusion of both the race effect, BF_inclusion_ = 0.41, and the interaction effect between race and task, BF_inclusion_ = 0.53. Examination of the descriptive statistics indicated that participants were faster at responding in the categorisation task (Mean (M) = 1666ms, Standard Deviation (SD) = 146.3ms) than they were in the individuation task (M = 1734ms, SD = 228.7ms).

The ANOVA on accuracy revealed extreme evidence that the task only model was preferred to the null, BF_10_ = 5.44^e+17^ and anecdotal evidence that the task only model was preferred to the full factorial model, BF = 1.36. Comparing effects across matched models revealed extreme evidence for the inclusion of the task effect, BF_inclusion_ = 6.43^e+17^, anecdotal evidence against the inclusion of the race effect, BF_inclusion_ = 0.23, and anecdotal evidence in favour of the inclusion of the interaction effect between race and task, BF_inclusion_ = 1.39. Examination of the descriptive statistics indicated that participants were more accurate when performing the categorisation task (M = 68.47%, SD = 9.26%) than when performing the individuation condition (M = 51.17%, SD = 7.2%).

#### Face specific region of interest analysis

In order to examine how variations in the amount and nature of contact with black people affected our participants’ response to black faces within the visual cortex an additional region of interest (ROI) analysis was conducted on the three clusters in the occipital cortex identified in the face > scrambled contrast using MarsBar^41^. Cluster wide parameter estimates from the contrasts between race (black > white) and task (categorisation > individuation) and the interaction between race and task ((black categorisation > white individuation) > (black categorisation > white individuation)) were extracted from each of the ROIs. The parameter estimates for each set of contrasts and for each of the three clusters were then entered into a series of Bayesian multiple regressions with default prior scales ^4^. For each regression the most complex model contained participants’ scores from the IAT, the measure of individuating experience, quality, quantity, cross-group friendship, and the positive and negative contact scales included as predictors. Table S1 reports the components of the best model containing any predictors, the Bayes Factor in favour of the best model compared to the null (BF_10_) and the R^2^ value of the best model. A BF_10_ of less than 1 indicates that the null model performed better than the best model containing any predictors.

**Table S1.** Cluster, contrast, best model, BF_10_ and R^2^ for each of the Bayesian regressions between contact variables and behavioural data. Indiv = individuating experience, PosCon = positive contact, NegCon = negative contact, CGF = cross-group friendship. * indicates moderate evidence in favour of the best model, ** indicates strong evidence in favour of the best model.

| Contrast | Cluster | Best Model | BF_10_ | R^2^ |
| --- | --- | --- | --- | --- |
| Race | rFG | Indiv | 0.668 | .067 |
|  | lIOG | IAT | 0.553 | .046 |
|  | lLOG | IAT | 0.447 | .023 |
| Task | rFG | PosCon + NegCon + Indiv | 1.72 | .312 |
|  | lIOG | NegCon | 1.303 | .135 |
|  | lLOG | NegCon | 0.55 | .046 |
| Interaction | rFG | PosCon + Indiv | 5.392* | .338 |
|  | lIOG | PosCon + Indiv + CGF | 12.239** | .461 |
|  | lLOG | PosCon + Indiv + IAT | 1.933 | .323 |

As can be seen in Table S1 only the regression analyses for the interaction contrasts on the rFG and lIOG regions produced models that had greater than anecdotal evidence over the null. Further analysis of the rFG interaction regression indicated that the best model contained the positive contact and individuating experience measures (see Table S2). This model had anecdotal support over the next best model which added the cross-group friendship measure to the other two terms, BF_01_ = 1.105. Inspection of the posterior summaries of coefficients indicated that positive contact had moderate support for inclusion and was positively correlated with the interaction term while individuating experience had anecdotal support for inclusion and was negatively correlated with the interaction term.

**Table S2.** Posterior summary of regression coefficients predicting size of parameter estimates extracted from the rFG interaction contrast. * indicates moderate evidence in favour of the best model

| Measure | β | Upper 95% Credible Interval | Lower 95% Credible Interval | BF_inclusion_ |
| --- | --- | --- | --- | --- |
| Positive contact | .147 | -.174 | 0.046 | 5.237* |
| Individuating contact | -.016 | -.03 | -.002 | 2.352 |

Further analysis of the lIOG interaction regression indicated that the best model contained the positive contact, individuating experience and cross group-friendship measures (see Table S3). This model had anecdotal support over the next best model which added the negative contact measure to the other three terms, BF_01_ = 1.44. Inspection of the posterior summaries of coefficients indicated that positive contact had moderate support for inclusion and was positively correlated with the interaction term, individuating experience had moderate support for inclusion and was negatively correlated with the interaction term and that cross group friendship had anecdotal support for inclusion and was positively correlated with the interaction term.

**Table S3.** Posterior summary of regression coefficients predicting size of parameter estimates extracted from the lIOG interaction contrast. * indicates moderate evidence in favour of the best model.

| Measure | β | Upper 95% Credible Interval | Lower 95% Credible Interval | BF_inclusion_ |
| --- | --- | --- | --- | --- |
| Positive contact | .145 | .049 | 0.049 | 6.633* |
| Individuating contact | -.028 | -.045 | -.011 | 5.07* |
| Cross-group Friendship | -.118 | -.016 | .252 | 1.569 |

# Supplementary References

1. Gelman, A., Hill, J. & Yajima, M. Why we (usually) don’t have to worry about multiple comparisons. *J. Res. Educ. Eff.* **5**, 189–211 (2012).

2. Sjölander, A. & Vansteelandt, S. Frequentist versus Bayesian approaches to multiple testing. *Eur. J. Epidemiol.* **34**, 809–821 (2019).

3. Rouder, J. N., Morey, R. D., Speckman, P. L. & Province, J. M. Default Bayes factors for ANOVA designs. *J. Math. Psychol.* **56**, 356–374 (2012).

4. Rouder, J. N. & Morey, R. D. Default Bayes factors for model selection in regression. *Multivariate Behav. Res.* **476**, 877–903 (2012).
